# 7-Diethyl­amino-2-oxo-2*H*-chromene-3-carbohydrazide

**DOI:** 10.1107/S1600536811010944

**Published:** 2011-04-13

**Authors:** Li-Jun Zhang, Bing-Zhu Yin

**Affiliations:** a Key Laboratory of Natural Resources of Changbai Mountain, & Functional Molecules (Yanbian University), Ministry of Eduction, Yanji 133002, People’s Republic of China

## Abstract

The asymmetric unit of the title compound, C_14_H_17_N_3_O_3_, contains two independent mol­ecules with different conformations of the ethyl groups. In the crystal, inter­molecular N—H⋯O hydrogen bonds link the mol­ecules into ribbons extending along the *a* axis.

## Related literature

For the bioactivity and chemiluminescence of coumarin derivatives, see: Munasinghe *et al.* (2007 ▶). For a related structure, see: Yu *et al.* (2009 ▶). For details of the synthesis, see: Ma *et al.* (2010 ▶).
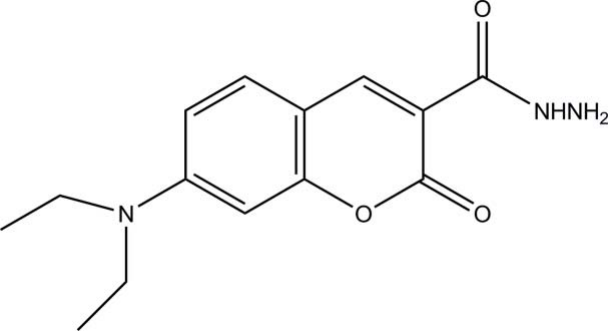


## Experimental

### 

#### Crystal data

C_14_H_17_N_3_O_3_
*M* *_r_* = 275.31
Triclinic, 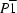

*a* = 9.3438 (19) Å
*b* = 12.771 (3) Å
*c* = 12.978 (3) Å
α = 95.17 (3)°
β = 110.13 (3)°
γ = 106.18 (3)°
*V* = 1366.4 (5) Å^3^
*Z* = 4
Mo *K*α radiation
μ = 0.10 mm^−1^
*T* = 290 K
0.14 × 0.12 × 0.11 mm

#### Data collection

Rigaku R-AXIS RAPID diffractometer
Absorption correction: multi-scan (*ABSCOR*; Higashi, 1995 ▶) *T*_min_ = 0.987, *T*_max_ = 0.990
13506 measured reflections
6186 independent reflections
3402 reflections with *I* > 2σ(*I*)
*R*_int_ = 0.030

#### Refinement

*R*[*F* ^2^ > 2σ(*F* ^2^)] = 0.045
*wR*(*F* ^2^) = 0.117
*S* = 1.01
6186 reflections
383 parameters
6 restraints
H atoms treated by a mixture of independent and constrained refinement
Δρ_max_ = 0.18 e Å^−3^
Δρ_min_ = −0.19 e Å^−3^

### 

Data collection: *RAPID-AUTO* (Rigaku Corporation, 1998 ▶); cell refinement: *RAPID-AUTO*; data reduction: *CrystalStructure* (Rigaku/MSC & Rigaku Corporation, 2002 ▶); program(s) used to solve structure: *SHELXS97* (Sheldrick, 2008 ▶); program(s) used to refine structure: *SHELXL97* (Sheldrick, 2008 ▶); molecular graphics: *PLATON* (Spek, 2009 ▶); software used to prepare material for publication: *SHELXL97*.

## Supplementary Material

Crystal structure: contains datablocks global, I. DOI: 10.1107/S1600536811010944/cv5061sup1.cif

Structure factors: contains datablocks I. DOI: 10.1107/S1600536811010944/cv5061Isup2.hkl

Additional supplementary materials:  crystallographic information; 3D view; checkCIF report

## Figures and Tables

**Table 1:** Hydrogen-bond geometry (Å, °)

*D*—H⋯*A*	*D*—H	H⋯*A*	*D*⋯*A*	*D*—H⋯*A*
N2—H2⋯O2	0.86 (3)	2.01 (2)	2.7098 (18)	137 (2)
N5—H5⋯O5	0.87 (3)	2.03 (2)	2.733 (2)	138 (2)
N6—H6*B*⋯O3	0.86 (3)	2.30 (1)	3.131 (2)	164 (2)
N3—H3*A*⋯O3^i^	0.87 (3)	2.20 (1)	3.002 (2)	155 (2)
N3—H3*B*⋯O6^ii^	0.87 (3)	2.23 (1)	3.039 (2)	154 (2)

Symmetry codes: (i) 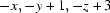; (ii) 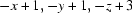.

**Fig. 1.**
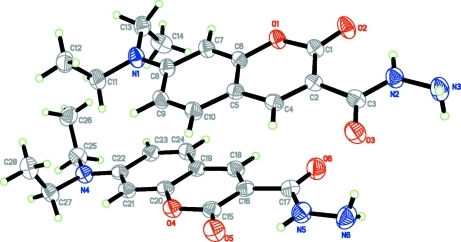
Two independent molecules of the title compound, with the atom numbering. Displacement ellipsoids of non-H atoms are drawn at the 30% probalility level.

## supplementary crystallographic information

### Comment

Coumarin derivatives have received considerable attention since their diverse bioactivities and chemiluminescence (Munasinghe *et al.* 2007). Herein, we report the crystal structure of the title compound, an important organic intermediate and a fluorescent tagging agent for chemosensors.

In the title compound, all bond lengths and angles are normal and comparable to those observed in the related struture (Yu *et al.*, 2009). There are two independent molecules in the asymmetric unit with different conformation of the ethyl groups (Fig. 1). Except for four terminal carbon atoms, the other non-hydrogen atoms are nearly coplanar for two components [mean deviations from the mean planes are 0.065 (1) and 0.07 (1), respectively] and form an angle of 19.39 (4) °. Intermolecuar N—H···O hydrogen bonds (Table 1) link the molecules into ribbons extended along axis *a*.

### Experimental

The title compound was prepared according to the literature (Ma *et al.*, 2010). Single crystals suitable for X-ray diffraction were prepared by slow evaporation a mixture of dichloromethane and petroleum (60–90 °C) at room temperature.

### Refinement

C-bound H-atoms were placed in calculated positions with C—H = 0.93, 096 or 0.97 Å and were included in the refinement in the riding model with *U*_iso_(H) = 1.2 or 1.5 *U*_eq_(C). The H of nitrogen atom were located from differecne Fourier Map and refined with N—H bond lengths restrained to 0.87 (3) Å, and with *U*_iso_(H) = 1.5 *U*_eq_(N).

### Crystal data

**Table:**

C_14_H_17_N_3_O_3_	*Z* = 4
*M**_r_* = 275.31	*F*(000) = 584
Triclinic, *P*1	*D*_x_ = 1.338 Mg m^−^^3^
Hall symbol: -P 1	Mo *K*α radiation, λ = 0.71073 Å
*a* = 9.3438 (19) Å	Cell parameters from 8202 reflections
*b* = 12.771 (3) Å	θ = 3.2–27.5°
*c* = 12.978 (3) Å	µ = 0.10 mm^−^^1^
α = 95.17 (3)°	*T* = 290 K
β = 110.13 (3)°	Block, yellow
γ = 106.18 (3)°	0.14 × 0.12 × 0.11 mm
*V* = 1366.4 (5) Å^3^	

### Data collection

**Table:**

Rigaku R-AXIS RAPID diffractometer	6186 independent reflections
Radiation source: fine-focus sealed tube	3402 reflections with *I* > 2σ(*I*)
graphite	*R*_int_ = 0.030
ω scans	θ_max_ = 27.5°, θ_min_ = 3.2°
Absorption correction: multi-scan (*ABSCOR*; Higashi, 1995)	*h* = −12→10
*T*_min_ = 0.987, *T*_max_ = 0.990	*k* = −16→16
13506 measured reflections	*l* = −16→16

### Refinement

**Table:**

Refinement on *F*^2^	Primary atom site location: structure-invariant direct methods
Least-squares matrix: full	Secondary atom site location: difference Fourier map
*R*[*F*^2^ > 2σ(*F*^2^)] = 0.045	Hydrogen site location: inferred from neighbouring sites
*wR*(*F*^2^) = 0.117	H atoms treated by a mixture of independent and constrained refinement
*S* = 1.01	*w* = 1/[σ^2^(*F*_o_^2^) + (0.0543*P*)^2^] where *P* = (*F*_o_^2^ + 2*F*_c_^2^)/3
6186 reflections	(Δ/σ)_max_ = 0.005
383 parameters	Δρ_max_ = 0.18 e Å^−^^3^
6 restraints	Δρ_min_ = −0.19 e Å^−^^3^

### Special details

**Table:**

Experimental. (See detailed section in the paper)
Geometry. All e.s.d.'s (except the e.s.d. in the dihedral angle between two l.s. planes) are estimated using the full covariance matrix. The cell e.s.d.'s are taken into account individually in the estimation of e.s.d.'s in distances, angles and torsion angles; correlations between e.s.d.'s in cell parameters are only used when they are defined by crystal symmetry. An approximate (isotropic) treatment of cell e.s.d.'s is used for estimating e.s.d.'s involving l.s. planes.
Refinement. Refinement of *F*^2^ against ALL reflections. The weighted *R*-factor *wR* and goodness of fit *S* are based on *F*^2^, conventional *R*-factors *R* are based on *F*, with *F* set to zero for negative *F*^2^. The threshold expression of *F*^2^ > σ(*F*^2^) is used only for calculating *R*-factors(gt) *etc*. and is not relevant to the choice of reflections for refinement. *R*-factors based on *F*^2^ are statistically about twice as large as those based on *F*, and *R*- factors based on ALL data will be even larger.

### Fractional atomic coordinates and isotropic or equivalent isotropic displacement parameters (Å^2^)

**Table:**

	*x*	*y*	*z*	*U*_iso_*/*U*_eq_	
C1	0.21139 (18)	0.57501 (13)	1.19989 (14)	0.0463 (4)	
C2	0.13466 (18)	0.48762 (12)	1.24574 (13)	0.0436 (4)	
C3	0.13127 (19)	0.50961 (13)	1.35921 (14)	0.0492 (4)	
C4	0.06725 (18)	0.38172 (12)	1.18533 (13)	0.0463 (4)	
H4	0.0150	0.3265	1.2146	0.056*	
C5	0.07300 (18)	0.35191 (12)	1.08046 (13)	0.0443 (4)	
C6	0.15295 (17)	0.43610 (12)	1.03866 (13)	0.0426 (4)	
C7	0.17389 (18)	0.41745 (13)	0.93993 (14)	0.0494 (4)	
H7	0.2289	0.4764	0.9159	0.059*	
C8	0.11153 (18)	0.30841 (13)	0.87521 (13)	0.0484 (4)	
C9	0.02840 (19)	0.22172 (13)	0.91645 (14)	0.0524 (4)	
H9	−0.0144	0.1489	0.8754	0.063*	
C10	0.01086 (19)	0.24390 (13)	1.01450 (14)	0.0516 (4)	
H10	−0.0443	0.1855	1.0391	0.062*	
C11	0.0769 (2)	0.17291 (15)	0.71424 (16)	0.0645 (5)	
H11A	0.0960	0.1239	0.7666	0.077*	
H11B	0.1411	0.1699	0.6698	0.077*	
C12	−0.0988 (2)	0.13259 (17)	0.63824 (16)	0.0768 (6)	
H12A	−0.1625	0.1375	0.6817	0.115*	
H12B	−0.1295	0.0565	0.6013	0.115*	
H12C	−0.1168	0.1780	0.5832	0.115*	
C13	0.2090 (2)	0.37670 (15)	0.72996 (15)	0.0640 (5)	
H13A	0.1828	0.4430	0.7462	0.077*	
H13B	0.1687	0.3532	0.6491	0.077*	
C14	0.3890 (2)	0.40375 (17)	0.77862 (19)	0.0777 (6)	
H14A	0.4301	0.4333	0.8577	0.116*	
H14B	0.4371	0.4580	0.7430	0.116*	
H14C	0.4149	0.3373	0.7662	0.116*	
C15	0.2466 (2)	0.11381 (14)	1.16812 (14)	0.0526 (4)	
C16	0.36577 (19)	0.22392 (14)	1.21170 (14)	0.0506 (4)	
C17	0.3901 (2)	0.29587 (15)	1.31736 (15)	0.0556 (4)	
C18	0.4607 (2)	0.26342 (14)	1.15444 (14)	0.0544 (4)	
H18	0.5359	0.3349	1.1819	0.065*	
C19	0.44932 (19)	0.20030 (13)	1.05563 (14)	0.0493 (4)	
C20	0.33527 (18)	0.09381 (13)	1.01525 (13)	0.0466 (4)	
C21	0.31367 (19)	0.02453 (13)	0.92049 (14)	0.0512 (4)	
H21	0.2345	−0.0454	0.8962	0.061*	
C22	0.41151 (19)	0.05924 (13)	0.85967 (14)	0.0494 (4)	
C23	0.5282 (2)	0.16798 (14)	0.90010 (14)	0.0547 (4)	
H23	0.5936	0.1939	0.8614	0.066*	
C24	0.5454 (2)	0.23412 (14)	0.99384 (15)	0.0571 (4)	
H24	0.6236	0.3045	1.0184	0.069*	
C25	0.4975 (2)	0.02687 (16)	0.70252 (16)	0.0687 (5)	
H25A	0.5055	−0.0386	0.6639	0.082*	
H25B	0.6054	0.0717	0.7547	0.082*	
C26	0.4354 (3)	0.09271 (17)	0.61840 (16)	0.0806 (6)	
H26A	0.3285	0.0490	0.5663	0.121*	
H26B	0.5055	0.1117	0.5786	0.121*	
H26C	0.4322	0.1596	0.6563	0.121*	
C27	0.2699 (2)	−0.11834 (14)	0.72177 (16)	0.0646 (5)	
H27A	0.2679	−0.1551	0.7837	0.078*	
H27B	0.2989	−0.1627	0.6727	0.078*	
C28	0.1023 (2)	−0.11651 (16)	0.65768 (16)	0.0717 (5)	
H28A	0.0664	−0.0811	0.7077	0.108*	
H28B	0.0299	−0.1915	0.6253	0.108*	
H28C	0.1040	−0.0758	0.5992	0.108*	
N1	0.12851 (17)	0.28688 (11)	0.77682 (12)	0.0596 (4)	
N2	0.19388 (19)	0.61432 (12)	1.41508 (12)	0.0581 (4)	
H2	0.231 (2)	0.6657 (13)	1.3829 (16)	0.087*	
N3	0.1989 (2)	0.64809 (13)	1.52280 (14)	0.0672 (4)	
H3B	0.268 (2)	0.6211 (18)	1.5658 (15)	0.101*	
H3A	0.1032 (15)	0.6189 (17)	1.5226 (19)	0.101*	
N4	0.39535 (17)	−0.00890 (12)	0.76634 (12)	0.0579 (4)	
N5	0.3063 (2)	0.25209 (14)	1.37695 (14)	0.0663 (4)	
H5	0.241 (2)	0.1841 (10)	1.3507 (18)	0.099*	
N6	0.3254 (2)	0.30988 (17)	1.48147 (15)	0.0782 (5)	
H6B	0.270 (3)	0.3544 (18)	1.470 (2)	0.117*	
H6A	0.4263 (14)	0.3504 (18)	1.5136 (19)	0.117*	
O1	0.21779 (13)	0.54484 (8)	1.09751 (9)	0.0493 (3)	
O2	0.27280 (15)	0.67378 (9)	1.24139 (10)	0.0638 (3)	
O3	0.07200 (17)	0.43205 (10)	1.39865 (11)	0.0697 (4)	
O4	0.23680 (13)	0.05352 (9)	1.07136 (9)	0.0542 (3)	
O5	0.15179 (15)	0.06693 (10)	1.20795 (11)	0.0694 (4)	
O6	0.48723 (17)	0.39132 (11)	1.34921 (11)	0.0751 (4)	

### Atomic displacement parameters (Å^2^)

**Table:**

	*U*^11^	*U*^22^	*U*^33^	*U*^12^	*U*^13^	*U*^23^
C1	0.0444 (9)	0.0409 (9)	0.0559 (10)	0.0098 (7)	0.0261 (8)	0.0083 (8)
C2	0.0416 (8)	0.0405 (8)	0.0540 (10)	0.0136 (7)	0.0244 (8)	0.0110 (7)
C3	0.0499 (9)	0.0471 (9)	0.0602 (11)	0.0185 (8)	0.0302 (8)	0.0131 (8)
C4	0.0442 (9)	0.0412 (9)	0.0556 (10)	0.0098 (7)	0.0238 (8)	0.0152 (8)
C5	0.0424 (8)	0.0398 (8)	0.0497 (9)	0.0092 (7)	0.0195 (7)	0.0114 (7)
C6	0.0362 (8)	0.0362 (8)	0.0508 (10)	0.0065 (7)	0.0163 (7)	0.0063 (7)
C7	0.0488 (9)	0.0415 (9)	0.0561 (10)	0.0046 (7)	0.0268 (8)	0.0080 (8)
C8	0.0442 (9)	0.0478 (9)	0.0495 (10)	0.0091 (8)	0.0199 (8)	0.0051 (8)
C9	0.0530 (10)	0.0374 (8)	0.0553 (11)	0.0043 (8)	0.0177 (8)	0.0028 (8)
C10	0.0530 (10)	0.0392 (8)	0.0568 (11)	0.0031 (8)	0.0236 (8)	0.0107 (8)
C11	0.0749 (13)	0.0558 (11)	0.0622 (12)	0.0167 (10)	0.0326 (10)	0.0004 (9)
C12	0.0884 (15)	0.0648 (12)	0.0565 (12)	0.0097 (11)	0.0179 (11)	0.0050 (10)
C13	0.0696 (12)	0.0645 (12)	0.0595 (12)	0.0189 (10)	0.0313 (10)	0.0050 (9)
C14	0.0777 (14)	0.0684 (13)	0.1055 (17)	0.0265 (11)	0.0537 (13)	0.0260 (12)
C15	0.0563 (10)	0.0527 (10)	0.0567 (11)	0.0170 (9)	0.0300 (9)	0.0196 (9)
C16	0.0521 (10)	0.0534 (10)	0.0534 (10)	0.0198 (8)	0.0252 (8)	0.0191 (8)
C17	0.0559 (10)	0.0583 (11)	0.0579 (11)	0.0219 (10)	0.0240 (9)	0.0202 (9)
C18	0.0549 (10)	0.0469 (9)	0.0607 (11)	0.0109 (8)	0.0250 (9)	0.0161 (9)
C19	0.0505 (9)	0.0470 (9)	0.0515 (10)	0.0100 (8)	0.0245 (8)	0.0168 (8)
C20	0.0456 (9)	0.0469 (9)	0.0530 (10)	0.0114 (8)	0.0265 (8)	0.0208 (8)
C21	0.0521 (10)	0.0441 (9)	0.0586 (11)	0.0078 (8)	0.0276 (9)	0.0168 (8)
C22	0.0505 (9)	0.0503 (9)	0.0535 (10)	0.0155 (8)	0.0261 (8)	0.0192 (8)
C23	0.0531 (10)	0.0576 (10)	0.0560 (11)	0.0089 (8)	0.0296 (9)	0.0208 (9)
C24	0.0576 (10)	0.0482 (10)	0.0611 (11)	0.0010 (8)	0.0297 (9)	0.0161 (9)
C25	0.0768 (13)	0.0694 (12)	0.0750 (13)	0.0238 (11)	0.0470 (11)	0.0165 (11)
C26	0.0798 (14)	0.0811 (14)	0.0630 (13)	0.0053 (12)	0.0223 (11)	0.0185 (11)
C27	0.0742 (13)	0.0515 (10)	0.0740 (13)	0.0163 (10)	0.0409 (11)	0.0064 (9)
C28	0.0660 (12)	0.0690 (12)	0.0765 (14)	0.0100 (10)	0.0366 (11)	0.0004 (10)
N1	0.0636 (9)	0.0501 (8)	0.0602 (9)	0.0039 (7)	0.0324 (8)	0.0005 (7)
N2	0.0738 (10)	0.0494 (9)	0.0568 (10)	0.0158 (8)	0.0362 (8)	0.0081 (7)
N3	0.0833 (12)	0.0614 (10)	0.0638 (11)	0.0183 (9)	0.0431 (10)	0.0042 (8)
N4	0.0605 (9)	0.0560 (9)	0.0631 (10)	0.0128 (7)	0.0359 (8)	0.0124 (8)
N5	0.0763 (11)	0.0713 (10)	0.0619 (10)	0.0225 (9)	0.0404 (9)	0.0155 (9)
N6	0.0926 (13)	0.0951 (14)	0.0573 (11)	0.0356 (11)	0.0380 (10)	0.0145 (10)
O1	0.0545 (6)	0.0380 (6)	0.0568 (7)	0.0050 (5)	0.0322 (6)	0.0062 (5)
O2	0.0802 (8)	0.0391 (6)	0.0750 (8)	0.0036 (6)	0.0481 (7)	0.0033 (6)
O3	0.0996 (10)	0.0517 (7)	0.0758 (9)	0.0195 (7)	0.0587 (8)	0.0168 (7)
O4	0.0581 (7)	0.0490 (6)	0.0606 (7)	0.0078 (5)	0.0360 (6)	0.0146 (6)
O5	0.0763 (9)	0.0634 (8)	0.0779 (9)	0.0078 (7)	0.0526 (8)	0.0166 (7)
O6	0.0871 (10)	0.0642 (9)	0.0697 (9)	0.0104 (8)	0.0380 (8)	0.0079 (7)

### Geometric parameters (Å, °)

**Table:**

C1—O2	1.2131 (19)	C16—C17	1.489 (2)
C1—O1	1.3757 (18)	C17—O6	1.231 (2)
C1—C2	1.443 (2)	C17—N5	1.332 (2)
C2—C4	1.354 (2)	C18—C19	1.406 (2)
C2—C3	1.487 (2)	C18—H18	0.9300
C3—O3	1.2372 (19)	C19—C20	1.393 (2)
C3—N2	1.324 (2)	C19—C24	1.406 (2)
C4—C5	1.403 (2)	C20—C21	1.367 (2)
C4—H4	0.9300	C20—O4	1.3808 (17)
C5—C6	1.395 (2)	C21—C22	1.410 (2)
C5—C10	1.404 (2)	C21—H21	0.9300
C6—C7	1.371 (2)	C22—N4	1.363 (2)
C6—O1	1.3785 (18)	C22—C23	1.422 (2)
C7—C8	1.409 (2)	C23—C24	1.350 (2)
C7—H7	0.9300	C23—H23	0.9300
C8—N1	1.3534 (19)	C24—H24	0.9300
C8—C9	1.425 (2)	C25—N4	1.475 (2)
C9—C10	1.353 (2)	C25—C26	1.496 (3)
C9—H9	0.9300	C25—H25A	0.9700
C10—H10	0.9300	C25—H25B	0.9700
C11—N1	1.462 (2)	C26—H26A	0.9600
C11—C12	1.502 (3)	C26—H26B	0.9600
C11—H11A	0.9700	C26—H26C	0.9600
C11—H11B	0.9700	C27—N4	1.462 (2)
C12—H12A	0.9600	C27—C28	1.507 (3)
C12—H12B	0.9600	C27—H27A	0.9700
C12—H12C	0.9600	C27—H27B	0.9700
C13—N1	1.487 (2)	C28—H28A	0.9600
C13—C14	1.501 (3)	C28—H28B	0.9600
C13—H13A	0.9700	C28—H28C	0.9600
C13—H13B	0.9700	N2—N3	1.406 (2)
C14—H14A	0.9600	N2—H2	0.86 (3)
C14—H14B	0.9600	N3—H3B	0.87 (3)
C14—H14C	0.9600	N3—H3A	0.87 (3)
C15—O5	1.2187 (18)	N5—N6	1.413 (2)
C15—O4	1.3732 (19)	N5—H5	0.87 (3)
C15—C16	1.445 (3)	N6—H6B	0.86 (3)
C16—C18	1.366 (2)	N6—H6A	0.87 (3)
			
O2—C1—O1	115.01 (14)	C16—C18—H18	118.7
O2—C1—C2	127.53 (15)	C19—C18—H18	118.7
O1—C1—C2	117.45 (14)	C20—C19—C18	117.92 (15)
C4—C2—C1	119.34 (14)	C20—C19—C24	116.24 (16)
C4—C2—C3	118.69 (14)	C18—C19—C24	125.83 (16)
C1—C2—C3	121.94 (14)	C21—C20—O4	116.70 (14)
O3—C3—N2	122.13 (15)	C21—C20—C19	123.23 (14)
O3—C3—C2	120.46 (15)	O4—C20—C19	120.07 (14)
N2—C3—C2	117.41 (14)	C20—C21—C22	119.95 (16)
C2—C4—C5	122.75 (14)	C20—C21—H21	120.0
C2—C4—H4	118.6	C22—C21—H21	120.0
C5—C4—H4	118.6	N4—C22—C21	121.31 (16)
C6—C5—C4	117.57 (14)	N4—C22—C23	121.43 (15)
C6—C5—C10	116.19 (14)	C21—C22—C23	117.25 (16)
C4—C5—C10	126.19 (14)	C24—C23—C22	121.02 (15)
C7—C6—O1	116.17 (13)	C24—C23—H23	119.5
C7—C6—C5	123.47 (14)	C22—C23—H23	119.5
O1—C6—C5	120.35 (13)	C23—C24—C19	122.30 (16)
C6—C7—C8	119.52 (15)	C23—C24—H24	118.9
C6—C7—H7	120.2	C19—C24—H24	118.9
C8—C7—H7	120.2	N4—C25—C26	113.65 (15)
N1—C8—C7	121.23 (15)	N4—C25—H25A	108.8
N1—C8—C9	121.16 (15)	C26—C25—H25A	108.8
C7—C8—C9	117.60 (14)	N4—C25—H25B	108.8
C10—C9—C8	120.88 (15)	C26—C25—H25B	108.8
C10—C9—H9	119.6	H25A—C25—H25B	107.7
C8—C9—H9	119.6	C25—C26—H26A	109.5
C9—C10—C5	122.33 (15)	C25—C26—H26B	109.5
C9—C10—H10	118.8	H26A—C26—H26B	109.5
C5—C10—H10	118.8	C25—C26—H26C	109.5
N1—C11—C12	111.83 (15)	H26A—C26—H26C	109.5
N1—C11—H11A	109.3	H26B—C26—H26C	109.5
C12—C11—H11A	109.3	N4—C27—C28	114.99 (15)
N1—C11—H11B	109.3	N4—C27—H27A	108.5
C12—C11—H11B	109.3	C28—C27—H27A	108.5
H11A—C11—H11B	107.9	N4—C27—H27B	108.5
C11—C12—H12A	109.5	C28—C27—H27B	108.5
C11—C12—H12B	109.5	H27A—C27—H27B	107.5
H12A—C12—H12B	109.5	C27—C28—H28A	109.5
C11—C12—H12C	109.5	C27—C28—H28B	109.5
H12A—C12—H12C	109.5	H28A—C28—H28B	109.5
H12B—C12—H12C	109.5	C27—C28—H28C	109.5
N1—C13—C14	111.26 (15)	H28A—C28—H28C	109.5
N1—C13—H13A	109.4	H28B—C28—H28C	109.5
C14—C13—H13A	109.4	C8—N1—C11	121.48 (14)
N1—C13—H13B	109.4	C8—N1—C13	122.05 (14)
C14—C13—H13B	109.4	C11—N1—C13	116.37 (13)
H13A—C13—H13B	108.0	C3—N2—N3	123.96 (15)
C13—C14—H14A	109.5	C3—N2—H2	118.5 (14)
C13—C14—H14B	109.5	N3—N2—H2	117.5 (14)
H14A—C14—H14B	109.5	N2—N3—H3B	103.8 (14)
C13—C14—H14C	109.5	N2—N3—H3A	108.3 (16)
H14A—C14—H14C	109.5	H3B—N3—H3A	112 (2)
H14B—C14—H14C	109.5	C22—N4—C27	121.26 (14)
O5—C15—O4	115.15 (15)	C22—N4—C25	121.55 (15)
O5—C15—C16	127.16 (16)	C27—N4—C25	117.10 (15)
O4—C15—C16	117.69 (14)	C17—N5—N6	122.83 (18)
C18—C16—C15	118.90 (16)	C17—N5—H5	117.4 (15)
C18—C16—C17	118.62 (16)	N6—N5—H5	119.7 (15)
C15—C16—C17	122.48 (15)	N5—N6—H6B	108.7 (17)
O6—C17—N5	121.62 (18)	N5—N6—H6A	106.1 (17)
O6—C17—C16	120.77 (16)	H6B—N6—H6A	107 (2)
N5—C17—C16	117.59 (17)	C1—O1—C6	122.45 (12)
C16—C18—C19	122.60 (17)	C15—O4—C20	122.81 (13)

### Hydrogen-bond geometry (Å, °)

**Table:**

*D*—H···*A*	*D*—H	H···*A*	*D*···*A*	*D*—H···*A*
N2—H2···O2	0.86 (3)	2.01 (2)	2.7098 (18)	137.(2)
N5—H5···O5	0.87 (3)	2.03 (2)	2.733 (2)	138 (2)
N6—H6B···O3	0.86 (3)	2.30 (1)	3.131 (2)	164 (2)
N3—H3A···O3^i^	0.87 (3)	2.20 (1)	3.002 (2)	155 (2)
N3—H3B···O6^ii^	0.87 (3)	2.23 (1)	3.039 (2)	154 (2)

Symmetry codes: (i) −*x*, −*y*+1, −*z*+3; (ii) −*x*+1, −*y*+1, −*z*+3.
